# Audiological evaluation in workers exposed to noise and pesticide

**DOI:** 10.1590/S1808-86942010000400003

**Published:** 2015-10-19

**Authors:** Heraldo Lorena Guida, Renata Graziele Morini, Ana Cláudia Vieira Cardoso

**Affiliations:** a PhD. Professor; b Speech and Hearing Therapist; c PhD. Professor

**Keywords:** hearing loss, pesticides, noise

## Abstract

Noise-induced hearing loss has been studied for many years and today many experts also investigate the synergic action of chemical products, since they can be potentially ototoxic.

**Aim:**

to investigate the audiological findings in workers exposed to occupational noise and pesticide and to compare it to data from noise-exposed workers.

**Study Design:**

Clinical retrospective.

**Material and Method:**

individuals that had been exposed to pesticide and noise (group I), and individuals that had been exposed to noise only (group II).

**Results:**

The classification of the audiometric findings showed in that group I: 35% had normal hearing thresholds, 53.75% had degree 1 hearing loss and 11.25% had degree 2 hearing loss; and group II had 57.5% of normal hearing, 40% had degree 1 hearing loss and only 2.5% had degree 2 hearing loss. The analysis of the audiometric findings also showed a significant worsening after comparing groups I and II thresholds, in the frequency of 3 kHz on the left ear and 4 kHz on both ears.

**Conclusion:**

The analysis showed that group I had worse audiometric thresholds compared to group II.

## INTRODUCTION

Thanks to technological development, the most spread type of pollution is sound pollution, and everyone is subject to being exposed to noise levels which are harmful for human health.

Noise, by itself, is harmful to health when the sound level is higher than 85 dB, depending on the duration and the systematic exposure to it. For this reason, based on this sound intensity, audiometries are periodically carried out in industrial plants^1^.

Noise induced hearing loss (NIHL) happens because of the systemic and prolonged exposure to intense noise, it is a chronic and irreversible disease, since it involves the hair cells of the organ of Corti. This disorder is also recognized by numerous authors as the most prevalent occupational-related disorder^2^.

NIHL initially affects the frequencies of 6, 4 or 3 kHz; and with the progression of the loss, it can reach the regions of 8, 2, 1 kHz, 500 and 250 Hz. Moreover, the individual can have tinnitus and discomfort related to intense sounds, and once the noise exposure ends, there is no more hearing loss progression. Exposure duration and individual susceptibility are also factors which can impact disease onset^3^.

When considering hearing loss in the lives of workers, it is very important to know other agents which can cause and/or worsen it - chemical products among them.

The first authors to discuss the interaction between noise and chemical products reported that the sensorineural hearing loss in workers exposed to solvents was more pronounced than expected in the case of noise exposure only^4^; this data was confirmed by another study^5^.

One study assessed the audiological profile of workers exposed to noise and chemical products in an alcohol and sugar plant. The authors classified the audiological findings^6^ and discovered that: 40% of the people working in the areas where they were exposed to chemical agents had hearing loss degree 1, making up the highest percentage of losses. In the area where there was exposure to noise and chemical agents, 10% of the workers had hearing loss degree 1 and 20% had it in degree 2 - showing a lower number of hearing loss, but a worsening in the degree, pointing to the expanded harmful effects of the associated agents. In the noise exposure area, 20% of them had hearing loss degree 1. They noticed the existence of a hearing hazard not only in the noise-exposed area, but also in those exposed to chemical products, thus indicating a greater severity when the exposure is associated^7^.

In a comparative study of audiometric exams from metallurgical workers exposed to noise (group I) and noise associated with chemical products (group II), we noticed that group II individuals had a higher prevalence of hearing loss when compared to those individuals in Group I^8^. Within this line of research, studies are being carried out with “insect busters” -professionals who control vectors by means of chemical products, mechanical devices or any other way used to remove the agents which are harmful to the population and still, are exposed to noise. These workers use pyrethroids and organophosphates insecticides^9^. These products were introduced in the group of high priority for studies associated with ototoxicity, in which we already had solvents, metals and asphyxiating substances^10^.

A recently held study with farmers exposed to organophosphates for five years concluded that such exposure increases the risk of the person developing hearing loss^11^.

Other authors have also shown central auditory dysfunction in workers using these types of insecticides in campaigns to fight vectors in endemies^12^.

A study carried out with workers exposed to noise and pyrethroids and organophosphates insecticides found that chronic exposure to these insecticides affect the peripheral and central auditory system, regardless of a concurrent exposure to noise^13^.

Another study analyzed audiometries from “insect busters” who worked regularly with pesticides in 2001/02, and in this latest year the company studied had 600 of these insect fighters. The study reported that 33 of these audiometric exams had NIHL-related alterations. Nonetheless, the authors argue that the study involved 37% of the workers; therefore, this figure can be three times greater. The study also challenged the hypothesis that these agents may have a synergic effect^9^.

An evaluation of the peripheral auditory alteration in a group of workers exposed to organophosphates and pyrethroid insecticides, used in control campaigns, showed that of the workers exposed to the insecticides, 63.8% had hearing loss. For the group with concurrent exposure to insecticides and noise, the hearing loss was of 66.7%. The average time taken to develop hearing alterations in the high mean frequencies, for combined exposures to insecticides and noise was 3.4 years, and for exposure to insecticides alone was 7.3 years. Hearing loss arising from concurrent exposure to both factors was higher in these frequencies than when the person was exposed to insecticides only^14^.

The investigation of the rural work process in nine towns in Minas

Gerais showed that about 50% of the workers interviewed were at least moderately affected by organophosphates and carbamates. This study shows the need for better controlling the exposure to these chemical agents^15^.

In a study held with farmers from Rio Grande do Sul, among the chemicals they were being exposed to there were pyrethroids and organophosphates, the authors found hearing loss in 60% of the individuals exposed to pesticides and noise, on the other hand, only 7% of the control group (without exposure to noxious elements) presented altered thresholds^16^.

When poisoning ensues, pesticides are classified as pyrethroids and act on the central and peripheral nervous system, and the high concentration cause permanent damage or long exposure damage the peripheral nerve. While those classified as organophosphates build up in tissues and when in excess they alter the central nervous system^17^.

Today it is rare to find corporate hearing protection programs which consider chemicals as possible culprits of auditory disorders, as well as its simultaneous exposure to noise. In 1999, in a very optimistic way, the Social Security Department (Decree # 3048 from May 06, 1999), listed benzene and aliphatic or aromatic hydrocarbonates as possible culprits of ototoxic hearing loss; nonetheless, this decree is limited to some solvents only^18^.

As to what was exposed above, some studies suggest that the exposure to two agents is really harmful to health, since they may have a synergic effect. Nonetheless, these studies are scarce and show a need for paying more attention to this matter, since there is a large number of Brazilians exposed to this hazard.

The goal of the present paper was to study the audiological findings in workers exposed to occupational noise and pesticides and compare them with data obtained from workers exposed to noise only.

## MATERIALS AND METHODS

This study was carried out in the clinical audiology department of a public institution. It is a retrospective study in which we analyzed the charts from individuals exposed to a pesticide (Malathion) and noise (group I), and individuals who were exposed to noise only (group II), in the year of 2007.

Each group was made up of 40 individuals, all male adults. These individuals were between 31 and 45 years, thus ruling out different diagnostic hypothesis, which happen after 45 years of life. And, in both groups noise exposure time was of 5 to 30 years.

The equivalent noise level that the workers were exposed to was 98.5 dB (A)9 and the exposure time was of 3 to 4 hours per day. The employees are previously fit with Personal Protection Equipment (P.P.E), according to Standard NR 620, of Ordinance 3214/781, being relevant for our study to stress the use of proper respiratory masks and ear plugs, both certified and approved by the Ministry of Employment and Labor.

The present study was approved by the Ethics in Research Committee (protocol # 2606/2007).

We studied information concerning the following procedures:

• audiological interview, in which we surveyed identification data and the auditory health history;
• threshold tonal audiometry, in which we assessed tonal thresholds of the subjects (air and bone conduction) ^19^;
• immittance measures, in which we analyzed data concerning tympanometry, with the aim of studying the functional integrity of the tympanic-ossicular chain in the auditory system^20^.

The audiometry tests were done in a sound-treated booth, using the GSI 61 Grason - Stadler audiometer. For acoustic immittance measures we used the GSI 38 Grason - Stadler immittance metering device.

Audiometric findings were analyzed and classified according to the occupational profile^6^ of the hearing loss.

In order to study the level of significance between the age of the individual and the time they had been working in that position we used the Student t-test. While the statistical analysis of the comparison of the audiologic results between groups I and II was carried out by the Anova test. In both cases the level of significance was 5% (p < 0.05) and the confidence interval was established at 95% of statistical confidence^21^.

## RESULTS

The results from the interview showed that the main complaints from the individuals in Group I were: tinnitus (n = 25 / 52.08%); allergies (n = 13 / 27.08%) and recruitment (n = 10 / 20.83%). In Group II, the main ones were: tinnitus (n = 21 / 58.33%); recruitment (n = 9 / 25 %) and autophonia (n = 6 / 16.66 %).

Among the data obtained from the anamneses, we plotted the age and duration of exposure to noise, which can be seen on Table 1. The data analysis show that there was no statistically significant difference between the mean values of age and duration of noise exposure, considering groups I and II.

**Table 1:** Analysis of the age and exposure duration between Groups I and II (t-Student Test).

Variable		Mean	SD	p value
Age (years)	Group I Group II	39,1 38,32	4,41 3,49	0,313^a^
Exposure time (years)	Group I	12,57	5,40	
	Group II	12,05	5,79	0,693^a^

SD: Standard Deviation

a non-significant result for a = 0.05

In assessing the functional integrity of the tympanic-ossicular chain, specifically in the tympanometry, we noticed that in Group I, both in the right ear and in the left ear, 35 (87.5%) individuals had type A curves and 5 (12.5%) had type Ad. While in Group II, 36 (90%) had type A tympanometric curve and 4 (10%) had type Ad, also bilaterally.

The results from the threshold tonal audiometries were analyzed from the frequencies of 500 Hz up to 8 kHz. In each frequency we analyzed the right and left ears of Group I compared to the right and left ears of Group II, in order to check whether or not the Anova statistical test would show significant differences between the groups. The data is presented on Table 2.

**Table 2:** Analysis of the tonal audiometry results, considering the mean values (dB HL) and the comparison between the groups I and II - Anova Test.

Frequency	Ear	Group I	Group II	p value
500 Hz	Right	9,87	9,37	0,706
	Left	7,0	8,25	0,355
1000 Hz	Right	8,62	8,87	0,886
	Left	7,25	8,5	0,416
2000 Hz	Right	8,5	8,25	0,884
	Left	10,87	8,5	0,159
3000 Hz	Right	15,75	12,62	0,240
	Left	19,5	12,37	0,008^b^
4000 Hz	Right	25,37	16,37	0,007^b^
	Left	25,37	18,87	0,023^b^
6000 Hz	Right	23,75	21,62	0,520
	Left	24,25	21,75	0,370
8000 Hz	Right	17,12	18,87	0,540
	Left	17,62	17,50	0,960

HLNA - hearing level

b significant difference for a = 0.05

**Graph 1:**
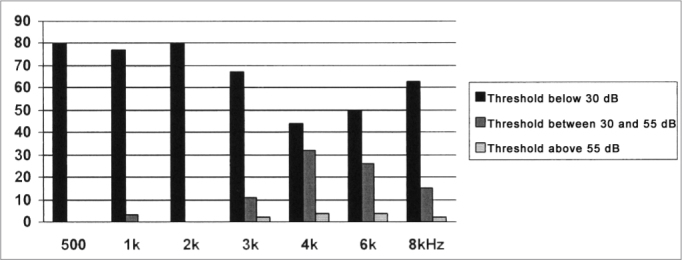
Distribution of the tonal threshold audiometry results for Group I, in the frequencies of 0.5 to 8 kHz.

**Graph 2:**
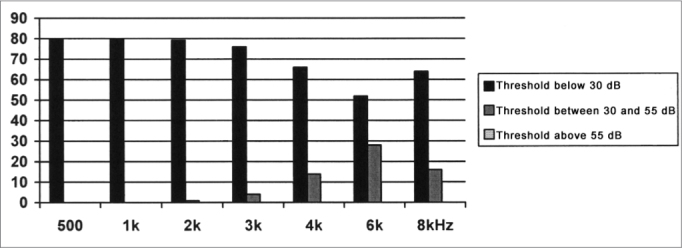
Distribution of the tonal threshold audiometry for Group II, in the frequencies of 0.5 to 8 kHz.

The results obtained from the threshold tonal audiometry, depicted on Graph 1, Graph 2, revealed that in both groups, in the frequencies of 0.5, 1 and 2 kHz the auditory thresholds were present in values below 30dB; therefore within the normal levels, bilaterally in most of the cases. While in the frequency range between 3 and 8 kHz, we observed a higher incidence of hearing loss at different levels.

When this value is compared between the groups, we can see a worsening in the auditory thresholds in the large majority of frequencies between 3 and 8 kHz, for group I. This worsening in the hearing loss was significant in the frequencies of 3 kHz (left ear) and 4 kHz (bilaterally), as per depicted on Table 2.

And finally, we did the analyses and classified the audiometries6. Thus, we noticed that in the right ear of Group I, 16 (40%) individuals were classified as normal, 20 (50%) had level 1 hearing loss and 4 (10%) had level 2 loss. While in the left ear of this same Group 12 (30%) were considered normal, 23 (57.5%) had level 1 loss, and 5 (12.5%) had level 2 loss. In Group II, we noticed that 23 (57.5%) individuals had normal hearing, 16 (40%) had level 1 loss and only 1 (2.5%) individual had level 2 hearing loss. These values were the same for the right and the left ears.

## DISCUSSION

In the present study, the most frequent complaints from Group I were tinnitus, allergy and recruitment; and for Group II they were: tinnitus, recruitment and autophonia. In both groups, we have the two complaints most frequently reported by the National Committee of Noise and Auditory Preservation^3^. Nonetheless, we have to stress the presence of the complaint of allergy in Group I, which can be associated with the contact with the pesticide.

The analysis of the immittance measures showed a predominance of type A curves, which matched the sensorineural profile of the noise induced hearing loss^20^. Even in the few cases with type Ad curve, we did not see any conduction problem in the audiometry.

In the present study, we see that in both groups the audiometries showed a hearing loss in the high frequencies, with peaks at 4 and 6 kHz, as it is commonly seen in audiometries from patients with NIHL^3^. This configuration showed that the lesion caused by the noise exposure and/ or pesticides reach a particular region of the cochlear basal turn. Other authors also reported a predominance of hearing loss in the high frequencies^7^^,^^8^^,^^13^.

In a quantitative analysis of the tonal threshold audiometry, a statistically significant difference was seen between the groups, with a worsening of the thresholds for group I, in the frequency of 3 kHz, in the left ear and in the frequency of 4 kHz, bilaterally.

In classifying the audiometries, we noticed that in Group I, more than 60% of the individuals have NIHL, while in Group II, this figure drops to 42.5%, in other words, the group with concurrent exposure to noise and pesticide presented a higher incidence of hearing loss, according to this classification^6^. Thus showing that both elements, working together, worsen the hearing loss. Therefore, the present study is in agreement with other scholars who discussed the interaction of noise and chemicals and found more pronounced hearing loss in those individuals exposed to both elements^4^^,^^5^^,^^7^^,^^8^^,^^13^^,^^14^^,^^15^.

## CONCLUSION

After analyzing the audiological findings and comparing Groups I and II, we conclude that the workers exposed to occupational noise and pesticide are under a higher risk of developing hearing loss when compared to those workers exposed to noise only. Thus, it is important that Hearing Protection Programs also consider these factors so as to better prevent the damages caused by these noxious elements.

## References

[1] Brasil. Ministério do Trabalho e Emprego. Portaria 3.214 de 8 de junho de 1978. Normas regulamentadoras de segurança e saúde no trabalho (NR-15): atividades e operações insalubres. Brasília, 1978. Disponível em http://www.tem.gov.br/legislacao/normas_regulamentadoras/nr_15.pdf. Acessado em 10 de junho de 2007.

[2] Ferreira JM. (1998). Perda auditiva induzida por ruído. Bom Senso e Consenso.

[3] (1994). Comitê Nacional de Ruído e Conservação Auditiva. Perda auditiva induzida pelo ruído relacionado ao trabalho. Acust Vibr..

[4] Barregard L, Axelsson A. (1984). Is there an ototraumatic interaction between noise and solvents?. Scand Audiol..

[5] Souza MMN, Bernardi APA. (2001). Ototoxidade dos produtos químicos: enfoque ocupacional. Rev Cefac..

[6] Merluzzi F, Parigi G, Cornacchia L, Terrana T. (1979). Metodologia di esecuzione del controllo delludito dei lavoratori esposti a rumore. Nuovo Arch Ital Otol..

[7] Fernandes T, Souza MT. (2006). Efeitos Auditivos em trabalhadores expostos a ruído e produtos químicos. Rev Cefac..

[8] Botelho CT, Paz APML, Gonçalves AM, Frota S. (2009). Estudo comparativo de exames audiométricos de metalúrgicos expostos a ruído e ruído associados a produtos químicos. Braz J Otorhinolaryngol..

[9] Vilela RAG, Malagolo ME, Morrone LC. (2005). Trabalhadores da saúde sob risco: o uso de pulverizadores no controle de vetores. Rev Prod..

[10] Morata TC. (2003). Chemical exposure as a risk factor for hearing loss. J Occup Environ Med..

[11] Crawford JM, Hoppin JA, Alavanja MC, Blair A, Sandler DP, Kamel F. (2008). Hearing Loss Among Licensed Pesticide Applicators in the Agricultural Health Study. J Occup Environ Med..

[12] Teixeira CF, Brandão MFA. (1998). Efeitos dos agrotóxicos no sistema auditivo dos trabalhadores rurais. Cad Inf Prev Acid..

[13] Teixeira CF. (2000). Exposição Ocupacional aos Inseticidas e seus Efeitos na Audição: A situação dos Agentes de Saúde Pública que Atuam em Programas de Controle de Endemias Vetoriais em Pernambuco [Dissertação].

[14] Teixeira CF, da Silva Augusto LG, Morata TC. (2002). Occupational exposure to insecticides and their effects on the auditory system. Noise Health.

[15] Soares W, Almeida RMVR, Moro S. (2003). Trabalho rural e fatores de risco associados ao regime de uso de agrotóxicos de Minas Gerais-Brasil. Cad Saúde Pública..

[16] Manjabosco CM, Morata TC, Marques JM (2004). Perfil Audiométrico de Trabalhadores Agrícolas. Arq Int Otorrinolaringol..

[17] Sucen. Segurança de Controle Químico de Vetores: Praguicidas. Disponível em http://www.sucen.sp.gov.br/saude_viajante/index.htm. Acessado em 25 maio de 2007.

[18] Azevedo, APM. Efeito de produtos químicos e ruído na gênese de perda auditiva ocupacional [dissertação]. Rio de Janeiro: Escola Nacional de Saúde Pública, Fundação Oswaldo Cruz. 2004.

[19] Momensohn-Santos TM, Russo I.P. (2005). Prática da Audiologia Clínica.

[20] Jerger J. (1970). Clinical experience with impedance audiometry. Arch Otolaryng..

[21] Levine DM, Berenson ML, Stephan D. (2000). Estatística: Teoria e Aplicações.

